# Protective genes and pathways in Alzheimer’s disease: moving towards precision interventions

**DOI:** 10.1186/s13024-021-00452-5

**Published:** 2021-04-29

**Authors:** Mabel Seto, Rebecca L. Weiner, Logan Dumitrescu, Timothy J. Hohman

**Affiliations:** 1 Vanderbilt Memory and Alzheimer’s Center, Vanderbilt University Medical Center, 1207 17th Ave S, Nashville, TN 37212 USA; 2 Vanderbilt Genetics Institute, Vanderbilt University Medical Center, Nashville, TN USA; 3 Department of Pharmacology, Vanderbilt University, Nashville, TN USA; 4 Department of Neurology, Vanderbilt University Medical Center, Nashville, TN USA

**Keywords:** Alzheimer’s, Genetic, Protection, Resilience

## Abstract

Alzheimer’s disease (AD) is a progressive, neurodegenerative disorder that is characterized by neurodegeneration, cognitive impairment, and an eventual inability to perform daily tasks. The etiology of Alzheimer’s is complex, with numerous environmental and genetic factors contributing to the disease. Late-onset AD is highly heritable (60 to 80%), and over 40 risk loci for AD have been identified via large genome-wide association studies, most of which are common variants with small effect sizes. Although these discoveries have provided novel insight on biological contributors to AD, disease-modifying treatments remain elusive. Recently, the concepts of resistance to pathology and resilience against the downstream consequences of pathology have been of particular interest in the Alzheimer’s field as studies continue to identify individuals who evade the pathology of the disease even into late life and individuals who have all of the neuropathological features of AD but evade downstream neurodegeneration and cognitive impairment. It has been hypothesized that a shift in focus from Alzheimer’s risk to resilience presents an opportunity to uncover novel biological mechanisms of AD and to identify promising therapeutic targets for the disease. This review will highlight a selection of genes and variants that have been reported to confer protection from AD within the literature and will also discuss evidence for the biological underpinnings behind their protective effect with a focus on genes involved in lipid metabolism, cellular trafficking, endosomal and lysosomal function, synaptic function, and inflammation. Finally, we offer some recommendations in areas where the field can rapidly advance towards precision interventions that leverage the ideas of protection and resilience for the development of novel therapeutic strategies.

**Supplementary Information:**

The online version contains supplementary material available at 10.1186/s13024-021-00452-5.

## Background

### Alzheimer’s disease

Alzheimer’s disease (AD) is a progressive, neurodegenerative disorder that is characterized by dementia, cognitive impairment in multiple cognitive domains, and an eventual inability to perform daily tasks. AD is the most common form of dementia and is distinguished by two main pathologies: beta-amyloid (Aβ) plaques and tau neurofibrillary tangles [1–3].

AD is often divided into two categories: early-onset AD (EOAD) and late-onset AD (LOAD). EOAD comprises only 1–5% of all AD cases and is classified by onset before the age of 65 [4, 5]. In contrast, the overwhelming majority of AD cases are late-onset and take place in individuals over the age of 65 [4, 6]. There are both sporadic and familial forms of EOAD and LOAD, where familial forms are most often associated with autosomal dominant mutations in genes such as *APP* (amyloid precursor protein), *PSEN1* (presenilin 1)*, PSEN2* (presenilin 2) [4, 5, 7]. However, sporadic forms of EOAD [8, 9] and LOAD have more complex etiology and are suggested to be polygenic [5, 10–14]. As the literature on familial EOAD and sporadic LOAD is more developed at this time, the scope of this review largely focuses on protection within these two subtypes.

Sporadic LOAD is multifaceted, with numerous environmental and genetic factors contributing to the disease [6]. LOAD is highly heritable with twin studies providing estimates of 60% < h^2^ < 80% [15], and to date over 40 risk loci for AD have been identified via large genome-wide association studies (GWAS), most of which are common variants with small effect sizes [16–18]. Although these discoveries have provided novel insight on the biological contributors to AD, disease modifying treatments for Alzheimer’s remain elusive [19–21].

### Protection and resilience

The ideas of resistance to pathology and resilience against the downstream consequences of pathology have been of particular interest in the AD field as studies continue to identify individuals with less than expected pathology, atrophy, or impairment given their age and/or neuropathological progression [22]. Protective factors can be defined as genetic [23] or environmental features [24] that reduce the risk that an individual will develop clinical AD. However, as our ability to measure the full neuropathological cascade of AD has expanded, the theoretical models have matured to include factors that protect from pathology, factors that protect against cognitive decline, and factors that protect against the downstream neurodegenerative cascade in AD (e.g., tau-related neurodegeneration) [25].

Resilience to AD, also known as asymptomatic or preclinical AD, is a phenomenon that in which individuals present with the neuropathological hallmarks of AD, but do not show clinical signs of cognitive impairment. In fact, as many as 70% of cognitively unimpaired older adults have some amount of AD pathology present in the brain at death, and as many as 30% of cognitively unimpaired older adults meet neuropathological criteria for autopsy-confirmed AD [26–28]. A shift in focus from AD risk to resilience presents an opportunity to uncover novel biological mechanisms of AD and to identify promising therapeutic targets for intervention. Such an approach has been transformative in other fields. For example, five loss-of-function variants in *PCSK9* that are associated with extremely low-density lipoprotein (LDL) cholesterol levels were identified in participants of the Dallas Heart Study [29]. These mutations led to the development of proprotein convertase subtilisin/kexin type 9 (PCSK9) inhibitors, which are currently used to treat statin-resistant hypercholesterolemia [21, 30]. In a similar way, uncovering and characterizing the genetic factors that protect against AD could lead to new therapeutic discoveries – in which pre-existing biological pathways could be modulated for treatment.

Protective factors contributing to resilience are broadly defined within the literature. In large genome-wide association studies looking at AD cases in comparison to controls, protective variant alleles and/or genes may be defined as those with odds ratio (OR) < 1 (as examples: [16, 17, 31, 32]). In studies using continuous outcomes, protective variants and/or genes may be defined as those associated with a delay in disease onset [33, 34] or those associated with less pathology than expected [35]. Additionally, protective genetic factors may arise through associations with known protective phenotypes such as longevity [36], cognitive reserve [37], educational attainment [38], or brain reserve [37, 39]. Cognitive reserve has been defined by Stern et al., [40] as the “adaptability of cognitive processes that helps to explain differential susceptibility of cognitive abilities to brain aging, pathology, or insult,” whereas brain reserve is described as the “neurobiological capital (i.e., number of neurons) that allows individuals to better cope with brain aging and pathology before clinical or cognitive changes arise [40].” Characterizing the manner in which genetic factors protect against AD is critical to advance the field. Genes may protect by reducing neuropathological burden, or by providing a more optimal response to high levels of neuropathology, or even by providing a higher biological or cognitive baseline that might buffer against the clinical manifestation of the first stages of AD [35]. In this review, we will carefully interrogate the evidence for emerging molecular pathways of protection and offer some recommendations for how the field can rapidly advance towards precision interventions that leverage this knowledge to develop novel therapeutic strategies.

### Inclusion criteria

**Fig. 1:**
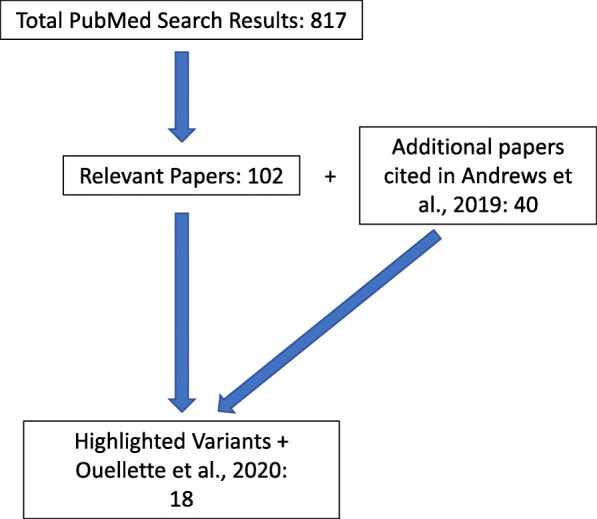
Summary of Literature Search and Results. A schematic demonstrating how search results were refined to those highlighted in the review

To identify AD protective or resilience variants and genes, we performed an initial PubMed search using the search terms: “protective variant Alzheimer”, “protective SNP Alzheimer’s Disease”, “protective GWAS Alzheimer”, and “SNP reduced risk AD”, which yielded a total of 817 search results. The search results were further filtered manually to those that were relevant and in scope of this review (Fig. 1). More specifically, we looked for previously identified variants in large GWAS and meta-analyses, case-control, cohort, or family studies, and rare variant analyses. Additionally, we included genes and variants that were previously reviewed or identified in the following papers: Andrews et al., 2019 [23] and Ouellette et al., 2020 [41]. Although many protective single nucleotide polymorphisms (SNPs) and genes have been identified within the literature (Supplemental Table 1), we have limited our discussion in this review to those with published functional evidence beyond genetic discovery analyses alone. “Functional evidence” includes (but is not limited to): additional analyses within the discovery manuscript, papers that replicated the original results, papers examining the biological effects of the variant of interest, papers that examine the annotated or referenced gene in the context of AD and referenced literature that help with interpretation and directionality of the biological mechanism behind protection.

#### Evidence categories

For each highlighted variant, the strength of evidence for their mechanism of action is varied; the categories may be defined: “modest”, “moderate”, and “strong.” For example, the mechanistic evidence for non-coding common variants may be limited to replication in different cohort studies. For “modest” evidence, we look to additional literature to suggest a putative functional gene near or within the locus and a biological pathway. Other variants may have “moderate” evidence, such that the functional gene within the region is well-established or that they cause an amino acid change within the encoded protein. For these variants, we look to literature to help us interpret directionality of effect and the biological pathway(s) behind protection. The “strong” genes and variants have all three facets of evidence (i.e., variant, gene, and defined mechanistic pathway); they have been replicated, they are annotated, and their function and mechanism of protection are well-studied. The level of evidence for each highlighted variant and gene is included in Table 1.

**Table 1:** Summary of Reviewed Variants and Genes

rsID^a^	Allele^b^	CPRA^b^	MAF^c^	Gene	Evidence	Reference
rs63750847	C > T	21:27269932:C:T	0.0001	*APP*	Strong	[42]
rs7412	C > T	19:45412079:C:T	0.087	*APOE*-ε2	Strong	[43]
rs121918393	C > A	19:45412013:C:A	0	*APOE3ch*	Moderate	[33]
rs9536314	T > G	13:33628138:T:G	0.147	*KL (*Klotho*-*VS*)*	Moderate	[44]
rs9527025	G > C	13:33628193:G:C	0.168	*KL (*Klotho*-*VS*)*	Moderate	[44]
rs10553596	T > −	10:115439641:T:-	0.19	*CASP7*	Moderate	[45]
rs2230806	C > T	9:107620867:C:T	0.29	*ABCA1*	Strong	[46]
rs72973581	G > A	19:1043103:G:A	0.05	*ABCA7*	Strong	[47]
rs11218343	T > C	11:121435587:T:C	0.052	*SORL1*	Moderate	[48]
rs142787485	A > G	2:26358156:A:G	0.0406	*RAB10*	Modest	[49]
rs3851179	T > C	11:85868640:T:C	0.361	*PICALM*	Modest	[50]
rs3796529	C > T	4:57797414:C:T	0.194	*REST*	Moderate	[51]
rs72824905	C > T	16:81942028:C:T	0.007	*PLCG2*	Moderate	[32]
rs3747742	T > C	6:41162518:T:C	0.306	*TREML2*	Moderate	[31]
rs1990621	C > G	7:12283873:C:G	0.447	*TMEM106B*	Modest	[52]
–	–			*MS4A* cluster	Modest	[53]
–	–			*BDNF*	Strong	[54]
–	–			*Dlgap2*	Moderate	[41]

*Abbreviations*: *CPRA* chromosome, position, reference allele, alternative allele, *MAF* minor allele frequency

^a^rsID is given for all variants except for reviewed genes whose wild-type forms are protective or for those in a multi-gene cluster (major > minor)

^b^Allele information from dbSNP (https://www.ncbi.nlm.nih.gov/snp/) and/or confirmed in the referenced literature

^c^Minor allele frequency information from dbSNP (ALFA project - global, https://www.ncbi.nlm.nih.gov/snp/docs/gsr/alfa/) and/or confirmed in the referenced literature

## Main text

**Fig. 2:**
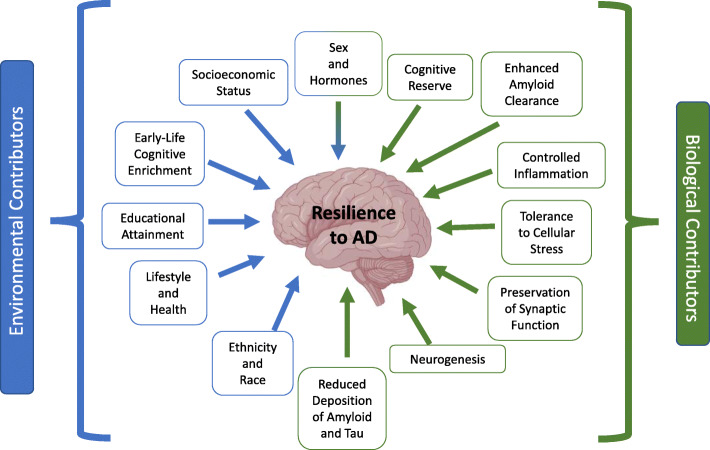
Theoretical Contributors to Resilience. A schematic demonstrating possible environmental and biological contributors to resilience to AD. The review focuses largely on proposed protective, biological pathways

Similar to AD risk, there are both protective biological and environmental contributors to resilience (Fig. 2). Our review focuses on a selection of genes and variants that directly mediate the cellular response to AD pathology or downstream cellular stressors of pathology in the brain. We discuss: APP [42], *APOE*-ε2 [55], *APOE*-ε3 Christchurch [33], *KL* [44], *CASP7* [45], *ABCA1* [46], *ABCA7* [47], *SORL1* [48], *RAB10* [49], *PICALM* [56, 57], *REST* [51], *BDNF* [54], *DLGAP1*, *DLGAP2* [41], *PLCG2* [32], *TREML2* [31], the *MS4A* gene cluster [53], and *TMEM106B* [52]*.* (Table 1). These genes also represent viable disease-modifying targets for AD, which could be modulated during and/or after pathological onset, but before cognitive impairment. Many of the genes and variants reviewed in this publication were initially identified in genome-wide association studies and meta-analyses of AD. A comprehensive list of protective variants and genes identified to date in such studies are included in Supplemental Table 1.

Common variants, especially those with a minor allele frequency (MAF) greater than 10%, can be very difficult to interpret in the context of risk and resilience. For example, one allele (often the minor allele) can be associated with protection from AD whereas the other allele (major) can be associated with risk. This interpretation is further complicated by minor allele flipping across populations. However, having variant, gene, and pathway level evidence can help aide our understanding of the biological mechanisms behind protective common variants.

When discussing protective variants within this review, the effective allele will be given in the text unless otherwise stated. In addition, the definitive mechanism of action may not be known for all common variants, so functional evidence is used to help interpret the acting gene within the region as well as the directionality of its mechanism of action.

### R.1 amyloid precursor protein A673T: reduced pathologic Aβ generation

*APP*, located on chromosome 21, is a gene that encodes amyloid precursor protein (APP). Aβ peptides are formed by the proteolytic cleavage of APP by α-, β-, and γ-secretases, and this processing pathway is also the source of neurotoxic Aβ, a major component of Alzheimer’s disease.

To date, there are over 60 identified mutations in the *APP* gene, with a large majority existing within coding regions [58]. Over 25 of these mutations are pathogenic and increase the risk of autosomal dominant Alzheimer’s disease through increasing Aβ production and oligomerization and reducing its clearance [58].

Though mutations in *APP* are often associated with an increased incidence of familial early-onset Alzheimer’s disease, Jonsson et al., identified a missense mutation within the *APP* gene in an elderly Icelandic population that was both protective against Alzheimer’s disease and associated with a slower decline in cognitive function among cognitively normal individuals [21, 42]. The identified variant is a rare single nucleotide polymorphism (SNP), rs63750847, that results in a substitution from alanine to threonine at position 673 of the protein (henceforth reported as p.A673T), which is near its β-secretase cleavage site [42]. Protection conferred by p.A673T is also further supported by in vitro studies demonstrating that the p.A673T allele results in a suboptimal β-secretase cleavage site that reduces production of pathologic Aβ by 50% in comparison to wild-type cells and delayed Aβ aggregation [59, 60]. In addition, a study within a Finnish male sample found that *APP* p.A673T carriers had 28% lower plasma levels of Aβ_40_ and − 42 compared to their age and *APOE* matched controls [61]. Altogether, there is strong gene- and pathway-level evidence that p.A673T’s is protective, and the data also suggest that a reduced amyloid burden throughout life is protective against AD.

However, the p.A673T allele is extremely rare [62–64], so it has not been verified whether it exhibits the same protective effect in non-Nordic populations. For example, the carrier frequency for p.A673T was only 0.018% in an US white population [63] and was found to be absent in a large Chinese sample [65] suggesting the protective effect of the allele may be limited to individuals of Nordic descent.

### R.1. Apolipoprotein E

The gene *APOE*, located on chromosome 19, encodes the protein Apolipoprotein E (APOE). There are three polymorphic alleles of *APOE*: *APOE*-ε2, ε3, and ε4, with estimated global allele frequencies of 8, 78, and 14%, respectively [66]. Well-established in the literature, *APOE-* ε4 is known as the greatest common genetic risk factor for AD [67–70], in which individuals carrying even one *APOE*-ε4 allele have up to 3 times increased risk for AD in comparison to ε3/ε3 homozygotes. Carrying two *APOE*-ε4 alleles can increase risk by up to 15-fold [71, 72].

In contrast, *APOE*-ε2 is considered to be a protective factor against AD [43, 73]. The magnitude of protection has been debated within the literature due to differences between neuropathologically- and clinically confirmed AD cases (i.e., individuals exhibiting clinical symptoms may be assigned to the AD group in a case-control study, though they do not meet neuropathological criteria for AD) [71]. A recent study with neuropathological samples by Reiman et al. demonstrated that the prevalence of AD was extremely low in *APOE*-ε2 homozygotes such that carriers of *APOE*-ε2 are 2.5 (ε2/ε3) to 8 (ε2/ε2) less likely to develop AD [71]. The proposed mechanism by which *APOE*-ε2 provides protection from AD is through reduced Aβ aggregation and improved Aβ clearance [73, 74]. However, the biological mechanisms underlying how *APOE*-ε2 enhances Aβ clearance have not yet been confirmed. One possible hypothesis of clearance is that APOE-ε2-Aβ complexes are more efficiently endocytosed and cleared within cells via their interaction with LDLR (low-density lipoprotein receptor), LRP1 (LDL receptor-related protein 1), and HSPGs (heparan sulfate proteoglycans), though this is still debated in the field [73–75]. Altogether, a reduction in brain amyloid levels appears to confer protection from AD.

In addition to Aβ clearance, recent literature suggests that *APOE* may also be involved in the spreading of tau downstream of amyloidosis. Arboleda-Velasquez et al. identified an individual with both an autosomal dominant AD mutation in *PSEN1* (presenilin 1, E280A) and two alleles of a rare mutation within *APOE*-ε3, called the Christchurch mutation (*APOE3ch*, p.R136S), who experienced a multi-decades-long delay in the onset of cognitive symptoms despite having widespread amyloid deposition throughout the brain as measured by PET. Although heterozygous individuals were present in the cohort, homozygosity of *APOE3ch* was required for protection. Interestingly, tau deposition (as measured by flortaucipir) was limited to the medial temporal and occipital lobes [33]. So far, these data suggest that the brain can withstand the widespread deposition of amyloid for a long period of time if tau deposition is limited before the onset of cognitive impairment.

Like *APOE*-ε2, *APOE3ch* displays similar protein-protein interactions with the LDLR and HSPG receptors, suggesting that it may offer protection through the same molecular mechanisms. For example, *APOE3ch* displays impaired binding affinity for HSPGs, and it has been suggested that this altered affinity may be responsible for its effects on tau deposition [33]. Recent studies by Therriault et al., examined the interaction of *APOE*-ε4 and Aβ on cerebrospinal fluid (CSF) and brain levels of tau supporting a probable relationship between APOE allele (e.g., *APOE3ch*) and tau deposition [76, 77]. This relationship is further supported by Shi et al., who demonstrated that of tau transgenic mice expressing *APOE*-ε4 had higher tau levels and more neurodegeneration than mice expressing *APOE*-ε2 or *APOE*-ε3 [78]. However, elucidation of the processes behind *APOE3ch*’s inhibition of tau spreading requires further study.

Given *APOE’s* involvement in AD, it has been explored as a potential therapeutic target for AD treatment. Anti-*APOE*-ε4 antibodies and antisense oligonucleotides that reduce brain APOE-ε4 levels have been explored, with positive results in reducing Aβ plaque burden [79, 80]. In addition, therapeutics that modulate APOE function to make it more “APOE-ε3-like” or “ε2-like” have been explored with relatively positive results in vitro and in murine models [79], though there are important considerations with regard to lipid health as homozygous ε2 carriers are likely to have a higher incidence of type III hyperlipoproteinemia [81]. However, efforts to target APOE therapeutically for AD have been somewhat limited due to its widespread expression throughout the body (i.e., brain and periphery) and its broad function in biological processes related to adipose function, fertility, and metabolism [82, 83]. A comprehensive review of *APOE* signaling in AD has been published previously, including the proposal of numerous therapeutic strategies [84]. The emergence of *APOE3ch* suggests that modifying APOE function and protein interactions (e.g., APOE-ABCA1, APOE-HSPG, APOE-Aβ) through antibodies or small molecules may be the most promising pathway for protection [84].

### R.2. Protection in the presence of APOE-ε4

As aforementioned, individuals carrying at least one copy of *APOE*-ε4 have significantly increased risk for AD and mortality [85]. However, not every *APOE*-ε4 carrier develops AD, suggesting that there are factors that confer protection in these higher-risk individuals [86]. Supporting this hypothesis, studies have identified variants that are protective from AD despite *APOE*-ε4 carriership.

An allele of the gene, Klotho (*KL*), named Klotho-VS was first implicated in human aging by Arking et al., in 2002 [87]. Klotho-VS is a haplotype containing two missense variants in linkage disequilibrium (LD): rs9536314 (p.F352V) and rs9527025 (p.C370S) [44]. Though it has been debated within the literature [88, 89], one allele of Klotho-VS has been associated with protective phenotypes such as: slower cognitive decline) [89, 90], greater cortical volume [91], and reduced amyloid burden [92]. Most recently, a study by Belloy et al., suggested that a single allele of Klotho-VS reduces AD risk by 1.3 times in *APOE-*ε4 carriers in comparison to *APOE-*ε4 carriers without Klotho-VS [44]. The authors also recapitulated previous findings that *APOE*-ε4-carrying Klotho-VS heterozygotes had reduced amyloid burden.

Klotho is involved in numerous biological functions, including growth-factor mediated signaling, calcium homeostasis, synaptic function, autophagy, cellular survival, and others [93, 94]. Higher levels of Klotho have been associated with longer life spans [95] and decreased markers of cellular aging (e.g., lower epigenetic age, higher telomerase activity) [96, 97]. Interestingly, heterozygotes with the -VS haplotype appear to have increased levels of Klotho and lower AD risk in comparison to homozygotes, suggesting that there is a protective range of Klotho [44]. At this time, there is no established connection between Klotho and APOE function in clearance, though Klotho appears to mediate amyloid clearance via autophagic pathways that interact with APOE [98–101]. Interestingly, Zhao et al. demonstrate that Klotho overexpression can reduce tau phosphorylation as well as improve Aβ clearance in a mouse model of AD, which suggests that Klotho can also reduce the neuropathological burden of amyloid and tau independently of APOE [102]. Though the evidence implicating Klotho-VS in AD is relatively strong, the exact biological pathway by which Klotho-VS is protective requires further study.

In another study identifying modifiers of AD risk in *APOE*-ε4 carriers, *APOE*-ε4 homozygotes carrying a common loss-of-function variant in *CASP7* (rs10553596) had roughly 2-fold reduced risk of AD compared to noncarriers [45]. rs10553596 represents a TT deletion within the coding region of *CASP7*; this causes both a leucine to serine amino acid change at position 44 of the protein as well as premature termination at position 133. Though caspase 7 is the likely functional gene, we can only speculate why this particular variant preferentially protects *APOE*-ε4 carriers. Caspase 7’s most well-established role is within the apoptotic cascade; however, it has been suggested that caspase 7 plays an integral role in the activation of microglia without initiating cell death [103]. Therefore, the loss of caspase 7 function may reduce aberrant microglial activation, thus limiting neuroinflammation, neurotoxicity, or cell death in response to pathology [103–105].

However, neither of the variants of KL (Klotho) or *CASP7* are protective in the absence of *APOE*-ε4, suggesting that the protective affects may only be seen under higher pathologic burden [44, 45]. Broadly, klotho-VS and caspase 7 (rs10553596) appear to exhibit protection via increasing cellular tolerance of stress [106, 107], though elucidation of their true therapeutic potential requires further examination. A major focus of the AD Sequencing Project (ADSP) Protective Variant Workgroup is to identify rare variants that provide protection among *APOE*-ε4 carriers, so exciting work in this space is on the horizon.

### R.3. Lipid signaling and homeostasis

*APOE* is also highly involved in lipid metabolism [82], and its major role in AD suggests that lipid signaling is an important etiological pathway of AD. Throughout the body, lipids play major roles in the structure and integrity of the cellular membrane, as well as endo- and exocytosis of macromolecules [108]. In the brain, studies suggest that they also play roles in blood-brain barrier function, inflammation, and myelination, among other processes [108]. Variants within lipid-related genes have been associated with both risk and resilience to AD, some of which include (but are not limited to) the following genes: *APOE* [84], *ABCA1* [109, 110]*, ABCA7* [111]**,** and *SORL1* [112]**,** which will be discussed further below. The protective SNPs identified within *SORL1*, *ABCA7*, and *ABCA1* further support a hypothesis that much of the genomic protection against AD relies on efficient clearance of pathology.

*ABCA1* encodes a protein of the same name (ABCA1, ATP-binding cassette transporter ABCA1) that mediates cholesterol efflux and APOE lipidation [113]. Two variants in ABCA1, rs2230805 and rs2230806, were identified as protective variants via a case-control study in a Hungarian sample [46]. Both rs2230805 and rs2230806 cause a non-synonymous amino acid change (p.L158L and p.R219K, respectively) and these SNPs are in strong linkage disequilibrium (LD, D′: 0.92; r^2^: 0.766) [46]. There has been some debate within the literature about whether rs2230805 and rs2230806 are truly protective [110]; however, there is evidence that the rs2230806/p.R219K variant delays the onset of LOAD by 1.7 years on average [34]. Functional studies suggest that ABCA1 deficiency increases Aβ deposition and exacerbates cognitive impairment in mice, especially in those expressing *APOE*-ε4 [114], so the protective effect may be mediated by increased expression of ABCA1 or a gain-of-function in ABCA1 protein leading to enhanced lipidation of APOE.

*ABCA7* (ATP-binding cassette transporter ABCA7) is also a gene within the same ATP-binding cassette transporter family [115]. A common variant in *ABCA7*, rs72973581 (Study MAF = 4.3%, [47]), results in a glycine to serine substitution at position 215 (p.G215S) and has been shown to reduce AD risk by roughly half [47]. Though *ABCA7* mediates lipid efflux and regulates lipid homeostasis similar to *ABCA1*, its protective effect appears to take a different path; *ABCA7* has a function in phagocytosis and APP processing [115]. For example, microglia from *Abca7*-deficient mice exhibit reduced capacity for phagocytosis and increased activation of β-secretase, resulting in higher levels of Aβ40 and 42 [116–118].

A protective variant was also identified within the *SORL1* (sortillin-related receptor 1) gene, which is a receptor for APOE [119]. More specifically, rs11218343-C is an intronic variant within *SORL1*, and the minor allele was associated with protection from AD in a genome-wide meta-analysis of Caucasian, Japanese, Korean, and Han Chinese individuals [48]. SORL1 is a member of the LDLR protein family as well as the vacuolar protein sorting 10 (VPS10) domain receptor family of proteins; it is suggested that SORL1 binds soluble Aβ and directs it to lysosomes for eventual degradation [120]. Though it is unclear how the minor allele rs11218343-C affects *SORL1* expression because it is in a non-coding region, *SORL1* loss-of-function or deficiency has been associated with AD [121–123]; therefore, a gain-of-function may be protective against AD. In addition, the gene-gene interaction between *APOE* and *SORL1* may also mediate amyloid clearance [124, 125]*.*

Similar to potential therapeutics that aim to increase the protective potential of APOE, targeting ABCA1, ABCA7, and SORL1 with activators (i.e., positive allosteric modulators, partial agonists, agonists) or increasing their expression may mimic the protective effect of the identified variants [126, 127]. Again, there are also *ABCA1*, *ABCA7*, and *SORL1* variants that increase the risk of AD [109–112], emphasizing the importance of lipid homeostasis in the neuropathological progression of AD. On the other hand, these protective variants suggest that lipid-mediated endocytosis and phagocytosis are important for amyloid clearance.

### R.4. Endosome/lysosome regulation

As aforementioned, lipid homeostasis is also connected to cellular trafficking [128]. Dysregulation of cellular trafficking (e.g., endosomal-lysosomal pathways, among others) has been associated with neurodegenerative disorders including AD [129], Parkinson’s disease, and amyotrophic lateral sclerosis [130], and variants within trafficking genes have been identified in large-scale GWAS and meta-analyses of AD [16–18]. From APP processing [131] and amyloid clearance [132] to neurotransmission [133], maintenance of cellular trafficking could both be a cause and/or consequence of mechanisms protecting individuals from AD.

*RAB10* (Ras-related protein Rab-10) encodes a protein of the same name that is a small GTPase and a key regulator of cellular trafficking [134]. The protective variant, rs142787485-G, is located in the 3′ untranslated region (UTR) of the *RAB10* gene and reduces AD risk by up to 1.7 times [49]. Though it is unclear whether the protective effect of rs142787485-G is through reduced *RAB10* expression, mRNA levels of *RAB10* are increased in AD, and there is evidence that RAB10 may also play a direct role in APP processing [49, 135]. In support of these hypotheses, in vitro studies demonstrate that shRNA-mediated knockdown of *RAB10* in mouse neuroblastoma cells results in a reduction of amyloid [49]. RAB10 has also been associated with the retromer complex, which mediates clearance of pathology [135]. However, RAB10 is also involved in other cellular functions such as the maintenance of endoplasmic reticulum (ER) morphology, axonogenesis, and neurotransmitter release, making it difficult to pinpoint its exact contribution to neuroprotection [135, 136].

A variant in the PICALM (phosphatidylinositol-binding clathrin assembly protein, PICALM) locus, rs3851179-A, exhibits protection from AD in numerous studies of European-decent (Caucasian/non-Hispanic white) participants (OR = 0.3–0.9) [56, 57]. However, it should be noted that this protective effect appears to be limited to *APOE*-ε4 noncarriers [50]. A clathrin-interacting protein, PICALM plays a major role in clathrin-mediated endocytosis, which can facilitate neurotransmission through receptor recycling and degradation [137, 138]. Variants in the PICALM locus have also been associated with increased risk of AD [139, 140], though the pathogenic mechanisms are still unclear. Ando et al. imply that PICALM is abnormally cleaved and downregulated in AD brains [141]. Other studies have suggested that PICALM modulates APP processing and Aβ clearance [138], and inducible pluripotent stem cell experiments have supported those findings [142]. Though the functional gene in the region has not been definitively demonstrated, these studies suggest rs3851179-A mediates protection through increased expression of *PICALM* and improved Aβ clearance, perhaps through endocytic mechanisms [142, 143].

Though the protective effects of the *RAB10* and *PICALM* variants appear to point toward APP processing and Aβ trafficking and clearance, both proteins also play important roles in synaptic function and neurotransmission. Therefore, RAB10 and PICALM may also implicate additional biological pathways that help preserve synaptic function in the presence of stressors such as AD pathology, as expanded upon in the next section.

### R.5. Synaptic dysfunction

Synaptic dysfunction is a hallmark of AD as well as many other neurodegenerative disorders, and it is believed to occur even before marked neurodegeneration and downstream cognitive impairment [129, 144]. Amyloid and tau burden are associated with synapse loss and dysfunction through both direct (i.e., tau-associated mitochondrial disruption) and indirect (i.e., neuroinflammation) pathways [144]. Synaptic plasticity is an important, biological correlate of learning and memory [145]; therefore, processes preserving synaptic density and function (even in the presence of pathology) are likely to be protective. There is notable genetic evidence of such protection from human genetic studies.

A transcriptional regulator, REST *(*restrictive element-1 silencing transcription factor), has been of interest with regard to neuronal development and brain aging. REST is a repressor of numerous genes including pro-apoptotic genes and others that mediate the cellular response to stress and to AD neuropathology [146]. In vitro, REST deficiency results in increased cellular damage and cell death relative to wild type, especially in response to cellular stressors such as hydrogen peroxide and Aβ [146]. Though REST expression in older adults (aged 73 to 106) is increased when compared to young adults (aged 20 to 35), its expression is significantly reduced in individuals with MCI and AD compared to controls [146]. A missense variant in exon 4 of *REST*, rs3796529-T, has been associated with slower hippocampal atrophy in individuals with MCI [51]. As REST mediates a wide array of biological processes, the effect mediated by rs3796529-T has not yet been confirmed. However, evidence suggests that higher levels of *REST* are beneficial due to its regulatory role in neurogenesis and neurodifferentiation as well as its ability to improve cellular tolerance to stress; therefore, rs3796529-T may result in a gain-of-function or an increase of *REST* expression [147, 148].

BDNF (brain-derived neurotrophic factor), an important protein for neural development, neurogenesis, and synaptic growth [149], is a downstream target of REST [150]. BDNF is also necessary for learning and memory [151], which is often impaired in AD; studies have suggested that BDNF is important for synaptic plasticity (such as long-term potentiation) in the hippocampus [151]. On average, individuals with AD have lower circulating levels of BDNF than controls, though there has been some debate within the literature [152]. In support of a protective role, Weinstein et al. demonstrated that higher levels of peripheral BDNF decreased AD risk, with the highest levels reducing risk by up to two-fold [54]. In addition, conditional BDNF expression in 5xFAD mice was able to rescue cognitive deficits and synaptic function [153]. Furthermore, BDNF overexpression was shown to be neuroprotective against amyloid in vitro [154] and was able to reduce Huntington-like phenotypes in mice [155].

A risk allele of BDNF has also been identified: rs6265-A or p.V66M [156, 157]. In addition to increased risk of sporadic AD, studies suggest that p.V66M increases the severity of cognitive decline, hippocampal atrophy, and neuropathological burden in autosomal dominant AD [158–160]. p.V66M negatively affects the secretion of BDNF [161], which supports the hypothesis that therapeutics increasing the efficacy, expression, or secretion of BDNF are expected to be protective.

The evidence for synaptic pathways also extends beyond human genomic discovery approaches. *Dlgap2* (disks large-associated protein 2) was recently identified as a protective candidate in a novel genetically diverse mouse model of AD and confirmed in a human GWAS [41]. Proteins within the DLGAP family, such as DLGAP2, function as important scaffolding proteins within the post-synaptic density and have been linked to neurological and psychiatric disorders including schizophrenia, AD, and Parkinson’s disease [162]. DLGAPs also play a role in modulating neuronal transmission though synaptic scaling [162]. Similar to BDNF, lower levels of DLGAP2 have been associated with AD as well as increased cognitive decline [41]. Additionally, a risk variant within DLGAP2 (rs6992443) was identified in a study examining the association of known epigenetically modified genes with LOAD [163]. Together, these data suggest that higher levels of DLGAP2 are likely to protect synaptic function. Another protein within the DLGAP family, DLGAP1 (also known as GKAP) is a nominated AD drug target on the Agora platform, which is a database of nominated targets for AD therapeutics, and increased expression is predicted to be protective, similar to DLGAP2 [164]. Aβ has been shown to mediate the degradation of DLGAP1 through phosphorylation by CDK5 [165]. Therefore, biological factors or therapeutics preventing the phosphorylation and/or degradation of DLGAP1 could help preserve synaptic function in the presence of pathology. Altogether, these variants and proteins support the idea that increased tolerance to cellular stress and continued maintenance of synaptic function are two interconnected mechanisms behind neuroprotection from AD and resilience.

### R.6. Immunity & inflammation

Neuroinflammation has been linked to overall pathophysiological changes within the brain during AD progression [166]. Microglia, the resident immune cells of the brain, are responsible in part for the clearance of amyloid through phagocytosis and the activation of additional immune cells. When the pathological burden in the brain is insurmountable by the immune system, inflammation becomes chronic and damaging to neurons due to the prolonged secretion of pro-inflammatory cytokines and factors by microglia [166]. Though many of the aforementioned protective variants primarily mediate amyloid clearance, variants that are able to modulate neuroinflammation (i.e., temper its damaging effects) are also likely to be protective. In addition, it should be noted that risk variants of *PLCG2* [167] and the *MS4A* gene cluster [168, 169] (discussed below) have been discovered.

*PLCG2* encodes phospholipase C gamma 2 (PLCγ2), which is expressed in microglia and granule cells within the brain [170]. A rare variant of *PLCG2* (rs72824905-G or P522R) reduces AD risk by nearly two-fold [32, 171]. PLCγ2 is a member of the phospholipase C-gamma family, and as such, cleaves phosphatidylinositol 4,5-bisphosphate (PIP_2_) into its products, inositol triphosphate (IP3) and diacylglycerol (DAG), that then propagate downstream signaling [172]. Though canonical phospholipid signaling serves a broad number of functions, PLCγ2 has been implicated in immune function and is believed to be in the same signaling pathway as *TREM2* [32], which has been identified as a genetic risk factor of AD [173]. The nonsynonymous amino acid change, p.P522R, appears to lie in a regulatory region of PLCγ2 and results in a hypermorphic form of the protein though the biological mechanism behind its neuroprotective effect is still unclear [170]. It should be noted, however, that increased inflammation is a double-edged sword; other gain-of-function mutations in PLCγ2 have been associated with autoimmune disorders [174].

Similar to *PLCG2*, *TREML2* (triggering receptor expressed on myeloid cell-like 2) is expressed by microglia [175, 176]. rs3747742-C (p.S144G) is a protective, missense coding variant within the *TREML2* gene [31]. Another protective, intergenic SNP between neighboring genes *TREM2* and *TREML2*, rs9381040, is in high LD with rs3747742 (D′: 0.86; r^2^: 0.67) and has a similar odds ratio as rs3747742 (OR = 0.92 and 0.93, respectively) [31]. rs3747742-C has been associated with lower levels of baseline CSF total tau (t-tau) as well as a slower rate of increase in CSF total tau levels, though there was no association with CSF levels of phosphorylated tau (p-tau) or amyloid [177]. In contrast, Benitez et al., demonstrate that rs3747742 and rs9381040 are both associated with lower levels of CSF p-tau, and their conditional analyses suggest that rs3747742 and rs9381040 represent the same signal [31]. TREML2 plays a pro-inflammatory role [175]; studies have shown that activated microglia and inflammatory cytokines are connected to tau pathology [178], suggesting that rs3747742-C reduces TREML2 activity though more studies are required to determine the exact mechanism by which the variant confers protection [177].

Another case-control study focusing on variants within the *MS4A* and *TREM* gene clusters demonstrated that a set of variants within the *MS4A* (membrane-spanning 4A) gene cluster were twice as frequent in controls than in AD cases [53]. Further investigation of the identified variants suggested that protection is conferred through a loss-of-function of MS4A family proteins [53], though additional studies are needed. However, MS4A genes have been previously associated with AD risk [168, 169]. Moreover, high levels of *MS4A6A* expression have been associated with elevated Braak scores [179]. There is also evidence that the *MS4A* locus plays a role in modulating *TREM2* expression*,* particularly soluble CSF TREM2 (sTREM2) levels. A GWAS of CSF soluble TREM2 (sTREM2) by Deming et al. suggested that protective *MS4A* gene cluster variants increased CSF sTREM2, which was associated with reduced AD risk and a delayed age-at-onset [180]. Together, these data functionally connect the *TREM2* and *MS4A* gene clusters and represent a potential mechanism by which inflammation can be modulated in the brain.

rs1990621-G, a variant within the *TMEM106B* (transmembrane protein 106B) locus, has been associated with neuronal protection in individuals with neurodegenerative disorders including AD [52]. rs1990621 is in high LD with rs3173615 (p.T185S, *r*^2^ = 0.98) [52], which was identified as a protective variant for frontotemporal lobar degeneration (FLTD), the second most-common cause of dementia in older adults [181]. rs1990621 is also in high LD with rs1990622 (*r*^2^ = 0.98) [52], which has been previously linked with familial, progranulin-related FLTD [182]. TMEM106B is a lysosomal protein that has been associated with aging and age-associated inflammation, and the risk alleles appear to be pro-inflammatory, perhaps through modulation of progranulin [183, 184]. However, the mechanism behind TMEM106B-mediated protection is unclear as TMEM106B expression is reduced in AD brains [181], but the risk alleles increase its mRNA expression in FLTD [185]. Altogether, TMEM106B-mediated protection from AD appears to be complex and requires further study.

AD drug discovery efforts have begun to include targets outside of amyloid and amyloid processing, with an increase in immune-modulating therapeutics. As of February 2020, 3 out of the 18 drugs in Phase 3 clinical trials have targeted inflammation with a focus on reducing neuroinflammation and increasing clearance of amyloid [186]. To date (November 2020), these trials are still ongoing. However, it is likely that the efficacy of an inflammatory-focused drug is dependent on the state (i.e., early/late) of disease [187, 188].

### Looking forward: precision medicine and quantitative measures of resilience

In this review, we have described gene variants that confer protection from AD or AD-associated phenotypes, even in the presence of *APOE-*ε4. Although other pathways of protection are represented in the literature, we focused on major biological processes that were implicated across multiple genetic studies of AD, including lipid metabolism, cellular trafficking, synaptic function, and inflammation. Several of the protective effects afforded by the variants appear to modulate brain amyloid levels and amyloid clearance, solidifying the role of amyloid in the disease progression of AD, though no anti-amyloid therapeutics have proven effective in treating cognitive impairment in clinical trials. The other protective mechanisms reviewed here include improved neuronal responses to stress (e.g., pathology or inflammation) and allow for the maintenance of synaptic homeostasis and function. Altogether, the protective processes converge on the cellular response to AD pathology. However, the vast majority of the studies that have identified protective variants rely on clinical phenotypes that cannot separate pathology from response to pathology. Thus, there remains an incredible opportunity to advance our understanding of protection through thoughtful analytical approaches that leverage the explosion of deep molecular biomarker data now available. To that end, we offer a few perspectives on how the field can rapidly advance towards precision therapeutics.

First, there is a pressing need for large genomic studies that integrate detailed metrics of neuropathology, neurodegeneration, and cognitive decline. For example, our team recently quantified a continuous measure of cognitive resilience by integrating established measures of amyloid pathology and harmonized measures of cognition [189]. Using these data, we identified variants upstream of the gene, *ATP8B1* (ATPase phospholipid transporting 8B1), that were associated with increased susceptibility to amyloid [189]. *ATP8B1* is an interesting candidate that encodes a protein by the same name that is important for modulating phospholipid composition within cellular membranes as well as maintaining bile acid homeostasis. Notably, deleterious variants were recently identified in another gene within the same family, *ATP8B4* (ATPase phospholipid transporting 8B4), via whole-exome sequencing [190], suggesting this family of flippases may be highly relevant to AD risk and progression. Although our study was the largest GWAS of resilience completed to date, we remained vastly underpowered to fully delineate the genetic architecture of resilience, highlighting the need for large-scale collaborative efforts to expand sample sizes and identify new signals. It is also notable that we did not observe a genetic correlation between clinical AD and resilience to AD, suggesting that genetic analyses exploring the downstream consequences of pathology will uncover novel molecular contributors to AD risk and protection.

In addition to the discovery of numerous common AD risk variants with low effect sizes, the failure of numerous anti-amyloid drugs in clinical trials have demonstrated that there is no singular variant, gene, or mechanism behind sporadic AD. Polygenic risk scores (PRS) that take the complexity of sporadic AD into account could be a useful way to predict an individual’s overall risk for disease. Recent studies have demonstrated the ability of PRS to predict AD with accuracy up to 84% [10]. PRS also present an exciting future for precision medicine as more genetic data are acquired and more risk loci are identified. Similar to PRS, a variant or gene with smaller effect size is unlikely to provide complete protection from AD on its own. As such, a “polygenic resilience score” combining both common and rare variants could not only help to predict individuals who are resilient from AD but could also provide new opportunities for AD drug discovery in the form of polypharmacology and/or pharmacogenetics.

It should also be mentioned that both risk and resilience conferred by common variants can vary across populations. For example, some studies have shown that APOE-ε4 alleles confer less AD risk in individuals of African descent than in non-Hispanic white individuals [191, 192]. However, African Americans are at increased risk of AD overall when compared to non-Hispanic whites [1, 193]. Though environmental differences between racial and ethnic groups (e.g., income, stress, discrimination) contribute to the pathogenesis of AD, a better understanding of the genetic architecture of AD in under-represented minority populations is scientifically and ethically critical to advance the field and enable personalized interventions.

Less than 30% of all published GWAS studies have focused on minority populations (e.g., individuals of African, Latin, or Hispanic descent), and in turn, most of what is known currently about AD genetic architecture is based on studies focusing on non-Hispanic white individuals [194]. Now, with technological advances and increased attention on healthcare disparities, the scientific field is working to increase representation in research studies [195–200].

Excitingly, emerging analyses within the past few years have not only identified novel AD risk loci in minority populations [196], but also have highlighted that risk loci in non-Hispanic white populations may not confer the same risk in groups of different race and ethnicity [191, 195]. Similar studies have also identified AD protective variants. Some notable examples are: rs75002042 (OR = 0.61), which is an intronic variant in the gene FBXL7 (F-box/LRR-repeat protein 7); it was identified in a case-control study of Caribbean-Hispanic individuals [197], and LRIG1 (Leucine Rich Repeats and Immunoglobulin Like Domains, OR = 0.54, rs2280575), which was discovered in an East Asian sample [199]. These findings, along with others, represent exciting advancements not only for minority populations but also for AD research.

Third, there is a growing literature on the genomics of educational attainment and cognitive performance that is relevant to cognitive reserve and protection from AD. In fact, educational attainment and cognitive performance are heritable [191–193]-- genetic differences can account for as much as 60% of the variation in educational attainment [194] and 70% of the variation in general cognitive ability [195, 196], which can be apparent even in early-life. Data from studies such as the Nun Study have demonstrated that early-life linguistic ability is associated with AD neuropathology and cognitive changes in late-life [197]. Furthermore, early-life cognitive enrichment (ELCE) was recently associated with slower age-related cognitive decline and late-life neuropathology [198], suggesting that intervention on modifiable risk factors at a young age affects performance in old age. The fact that much of the cognitive benefits of ELCE were independent of AD neuropathology suggests there are distinct and complex pathways that promote resilience (i.e., pathology-related versus pathology-independent) [199]. Fully encompassing the genetic architecture of cognitive ability into our models of AD resilience will be critical as we move to better understand the molecular pathways that protect against AD.

Fourth, AD is a disease of aging, and the strongest genetic risk factor for the disease (*APOE*) has a robust association with longevity [200, 201]. Far more work integrating the genetic architecture of longevity related traits into our models of AD are needed to better understand how these pathways intersect. For example, telomere length is strongly associated with life span, and shortened telomeres are indicative of cell aging [202]. In 2020, a drug to lengthen telomeres through transduction of human TERT (telomerase reverse transcriptase) was in Phase I clinical trials [186]. However, the direction of telomere effects, the relevant cell types, and changes over the course of age and disease remain poorly understood, providing a critical knowledge gap for future work [203]. Similarly, disentangling the effects of longevity genes on survival from the effects on neuropathological burden and age-related cognitive decline will be critical to better understand and prioritize molecular pathways that contribute to longevity and AD.

Finally, there is an incredible opportunity to advance our understandings of protection by focusing on the notable heterogeneity in the neuropathological presentation and clinical manifestation of the disease across sexes. Nearly two-thirds of diagnosed AD cases are women [1, 204] and *APOE*-ε4 is more strongly associated with clinical AD [205] and measures of tau [206]. Moreover, AD neuropathology is more likely to clinically manifest as clinical dementia in women than in men [207, 208]. There is now emerging work implicating sex-specific genomic and transcriptomic signatures of AD in humans, and work in mouse models has implicated the important contribution of both gonadal hormone and X-chromosome effects on conferring risk and resilience to AD in a sex-specific manner. Yet, the vast majority of studies of protection in AD have not integrated sex-specific models, and the degree to which the molecular contributors to resilience differ by sex remains poorly understood [206, 207, 209–214]. Further exploration into sex differences in biological mechanisms driving resilience to AD could present a turning point for precision medicine by clarifying whether the best target pathway for intervention varies by age, biomarker status, genetic background and sex [215].

## Conclusions

Sporadic AD presents immense therapeutic challenges due to the heterogeneity in the neuropathological presentation, age of onset, rate of decline, and clinical manifestation of disease. However, this same heterogeneity provides an exciting opportunity to characterize the specific molecular context in which neuroprotection is observed. The powerful stories of protection in even a single high-risk patient can transform our molecular understanding of a disease. The new identification of a protected autosomal dominant mutation carrier has provided exciting new directions for AD therapeutics, and we must find a way to identify such incredible stories of resilience in sporadic AD that surely are hiding in our ever-expanding cohort studies of aging and AD.

## Supplementary Information

**Additional file 1.**

## Data Availability

Not Applicable.

## Abbreviations

Aβ: Beta-amyloid
AD: Alzheimer’s disease
ADSP: AD Sequencing Project
CSF: Cerebrospinal fluid
ELCE: Early-life cognitive enrichment
EOAD: Early-onset Alzheimer’s disease
FLTD: Frontotemporal lobar degeneration
GWAS: Genome-wide association study
h^2^: heritability
LD: Linkage disequilibrium
LDL: Low-density lipoprotein
LOAD: Late-onset Alzheimer’s disease
MAF: Minor allele frequency
OR: Odds ratio
p-tau: phosphorylated tau
PET: Positron emission tomography
PRS: Polygenic risk score
SNP: Single nucleotide polymorphism
t-tau: total tau
